# Distribution and dynamics of *Anopheles gambiae* s.l. larval habitats in three Senegalese cities with high urban malaria incidence

**DOI:** 10.1371/journal.pone.0303473

**Published:** 2024-05-14

**Authors:** Fatou Ndiaye, Abdoulaye Diop, Joseph Chabi, Katherine Sturm-Ramirez, Massila Senghor, El Hadji Diouf, Badara Samb, Seynabou Mocote Diedhiou, Omar Thiaw, Sarah Zohdy, Ellen Dotson, Doudou Sene, Mame Birame Diouf, Valerie Koscelnik, Lilia Gerberg, Abdoulaye Bangoura, Tiffany Clark, Ousmane Faye, Ibrahima Dia, Lassana Konate, El Hadji Amadou Niang

**Affiliations:** 1 Laboratoire d’Ecologie Vectorielle et Parasitaire, Université Cheikh Anta Diop de Dakar, Dakar, Sénégal; 2 U.S. President’s Malaria Initiative VectorLink Project, Dakar, Senegal; 3 U.S. PMI VectorLink Project, Abt Associates, Rockville, MD, United States of America; 4 U.S. President’s Malaria Initiative Dakar, Dakar, Senegal; 5 U.S President’s Malaria Initiative, Centers for Disease Control and Prevention (CDC), Atlanta, GA, United States of America; 6 National Malaria Control Programme, Dakar, Senegal; 7 U.S. President’s Malaria Initiative, United States Agency for International Development (USAID), Washington, DC, United States of America; 8 Institut Pasteur de Dakar, Unité d’Entomologie Médicale, Dakar, Senegal; University of Glasgow College of Medical Veterinary and Life Sciences, UNITED KINGDOM

## Abstract

Urban malaria has become a challenge for most African countries due to urbanization, with increasing population sizes, overcrowding, and movement into cities from rural localities. The rapid expansion of cities with inappropriate water drainage systems, abundance of water storage habitats, coupled with recurrent flooding represents a concern for water-associated vector borne diseases, including malaria. This situation could threaten progress made towards malaria elimination in sub-Saharan countries, including Senegal, where urban malaria has presented as a threat to national elimination gains. To assess drivers of urban malaria in Senegal, a 5-month study was carried out from August to December 2019 in three major urban areas and hotspots for malaria incidence (Diourbel, Touba, and Kaolack) including the rainy season (August-October) and partly dry season (November–December). The aim was to characterize malaria vector larval habitats, vector dynamics across both seasons, and to identify the primary eco- environmental entomological factors contributing to observed urban malaria transmission. A total of 145 *Anopheles* larval habitats were found, mapped, and monitored monthly. This included 32 in Diourbel, 83 in Touba, and 30 in Kaolack. The number of larval habitats fluctuated seasonally, with a decrease during the dry season. In Diourbel, 22 of the 32 monitored larval habitats (68.75%) were dried out by December and considered temporary, while the remaining 10 (31.25%) were classified as permanent. In the city of Touba 28 (33.73%) were temporary habitats, and of those 57%, 71% and 100% dried up respectively by October, November, and December. However, 55 (66.27%) habitats were permanent water storage basins which persisted throughout the study. In Kaolack, 12 (40%) permanent and 18 (60%) temporary *Anopheles* larval habitats were found and monitored during the study. Three malaria vectors (*An*. *arabiensis*, *An*. *pharoensis* and *An*. *funestus* s.l.) were found across the surveyed larval habitats, and *An*. *arabiensis* was found in all three cities and was the only species found in the city of Diourbel, while *An*. *arabiensis*, *An*. *pharoensis*, and *An*. *funestus* s.l. were detected in the cities of Touba and Kaolack. The spatiotemporal observations of immature malaria vectors in Senegal provide evidence of permanent productive malaria vector larval habitats year-round in three major urban centers in Senegal, which may be driving high urban malaria incidence. This study aimed to assess the presence and type of anopheline larvae habitats in urban areas. The preliminary data will better inform subsequent detailed additional studies and seasonally appropriate, cost-effective, and sustainable larval source management (LSM) strategies by the National Malaria Control Programme (NMCP).

## Introduction

Global malaria control interventions have led to a significant decline in malaria-related morbidity and mortality worldwide. According to the 2021 world malaria report, between 2000 and 2019 the global incidence of malaria declined from 81 to 56 cases per 1000, though an upsurge of malaria cases has been documented since 2020 following the disruption of essential health services due to the COVID-19 pandemic [1]. To maintain gains towards malaria elimination, continuous strengthening and adaptation of existing control efforts to target unique contexts, such as malaria in urban settings, is necessary.

Urbanization and population growth have been shown to impact urban living conditions, proliferating mosquito larval habitats through flooding, construction, water storage, and inappropriate drainage, increasing malaria cases in cities and transforming urban malaria into an emerging public health threat in Africa [2–7]. African cities have grown over the past decade and about 40% of the population in the ten highest burden malaria endemic countries in Africa are now reported to live in urban areas [8–11]. As such, it is critical to investigate the driving factors of malaria transmission to better understand its epidemiology in urban settings, and design targeted, adapted interventions which may differ from those in the rural context [12–14].

In Senegal, urban malaria conditions have been described as a threat to the country’s elimination efforts [5,8,11]. Through its national strategic plan (NSP 2016–2020), Senegal committed to accelerating malaria control to reach the epidemiological threshold of pre-elimination [15]. Within the framework of the 2016–2020 NSP, different interventions were implemented by zone, according to malaria incidence [15]. To achieve its goals, a malaria control action plan was strategically oriented towards targeting interventions according to epidemiological characteristics. Thus, indoor residual spraying (IRS) and mass distribution of insecticide treated nets (ITNs) were implemented across the country to protect populations at risk for malaria, particularly children under 5 years of age and pregnant women [16,17].

The implementation and scale-up of both IRS and ITNs along with other preventive and case management measures contributed to the decline of malaria in Senegal [18]; however, malaria remains an important public health problem, especially in urban settings, where spatio-microecological dynamics of malaria transmission are influenced by natural (flooding, presence of rivers or mangrove swamps) and anthropic factors (urbanization, irrigation) which increase the presence of vector larval habitats[5–7,8,11,19–22]. In 2016 and 2017, Diedhiou *et al*. demonstrated a strong association between high urban malaria incidence and flooding and subsequent proliferation of *Anopheles* larval habitats in the suburbs of the capital Dakar [23,24]. To better understand the drivers of high malaria incidence observed in urban areas, a deeper assessment of the seasonal persistence and productivity of vector larval habitats in urban areas is needed.

The present study was conducted between August to December 2019 in Diourbel, Touba, and Kaolack to assess the presence and type, and characterize the anopheline larvae habitats in urban areas to better guide evidence-based tailored, cost-effective, and sustainable larval source management in eligible urban areas.

## Materials and methods

### Ethics statement

This study was done under the lead of the Senegalese NMCP and focused on *Anopheles gambiae* s.l. larval habitats distribution, occupancy, and dynamics. It does not involve harmful activities or endangered or protected species and thus does not require any ethics approval from an authority or consent to participate. The individuals pictured in Fig 4 have provided written informed consent (as outlined in PLOS consent form) to publish their images alongside the manuscript.

### Study sites

**Fig 1:**
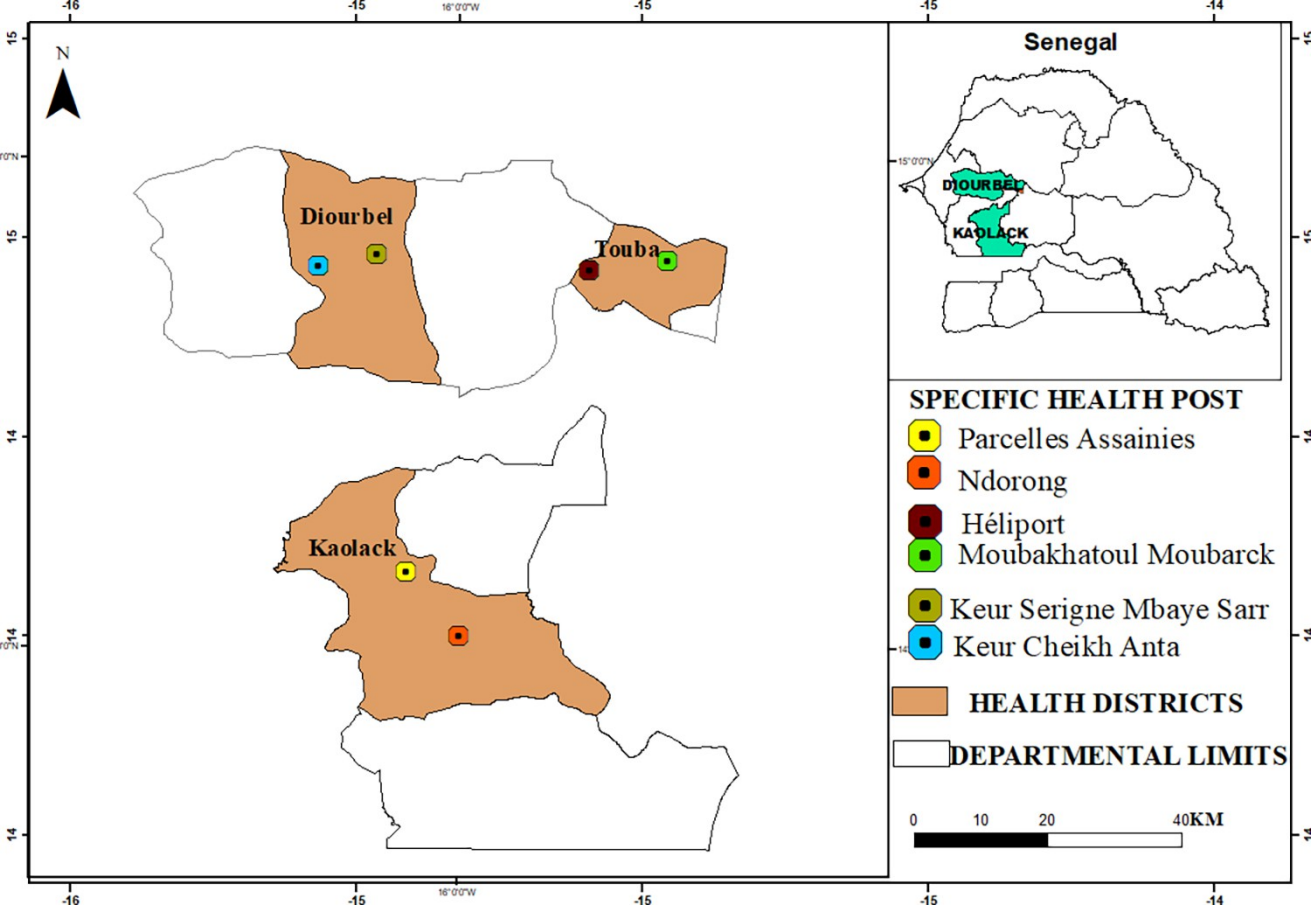
Geographical location of the study area, health districts, and health posts (Copyright © 1995–2021 Esri. All rights reserved. Published in the United States of America.).

The study was conducted in the cities of Diourbel, Touba and Kaolack in central-western Senegal. The three study cities are the most populous cities in Senegal after the capital city of Dakar, facing recurrent flooding and correspond to hotspots of high malaria incidence and increasing human population density (Fig 1).

Diourbel (14°39′18″N, 16°13′53″W), 145 km away from Dakar, is administratively subdivided into four districts (Diourbel, Touba, Bambey and Mbacké). With an average temperature above 40˚C, average annual rainfall of 485.9 mm, and an estimated population of 1,919,090, Diourbel is the only region in Senegal characterized by the presence of a perennial river with an ancient fossil valley, creating favorable conditions for crop cultivation throughout the year. Malaria remains endemic in the region, with an incidence of 9.2 cases per 1000 inhabitants in 2020 [22]. In the city of Diourbel, the study was conducted in the neighborhoods of Keur Serigne Mbaye Sarr and Keur Cheikh Anta and extended to some of their suburbs, namely Thierno Kandji, Médinatoul Nord, Keur Cheikh Ibra, and Cité Ouvriére, because of ecological characteristic similarities with the sentinel localities.

The city of Touba (14° 51′ 00″ N, 15° 53′ 00″ W) is about 194 km away from Dakar and represents the second most populated city of Senegal after the capital, with 906,514 inhabitants settled in an area of 120 km^2^. Although the temperature and the average rainfall are similar to Diourbel, Touba represents the district with the highest malaria incidence of the three districts with 14.0 cases/1000 inhabitants in 2020 [22]. Furthermore, Touba is one of the religious centers of Senegal with annual population fluctuations, especially during “*The Grand Magal of Touba*”. During this time, the location is also considered to be a critical hub of population movement with seasonal migration from across borders and across the country to this location. In Touba, the neighborhoods of Héliport and Boukhatoul Moubarack, located at the periphery of the city, were the study sites selected (Fig 1).

Kaolack (14° 10′ 00″ N, 16° 05′ 00″ W), located 192 km away from Dakar, has a population estimated at about 380,010 inhabitants during the latest census in 2019. The city is the heart of the groundnut growing basin and located at the crossroad of trans-Gambia with agriculture and trading as the main activities within the area. The city is characterized by a poor water drainage system with open canals and valleys, representing adequate mosquito larval habitats. The average rainfall is 776 mm and malaria incidence of 8.7 cases/1000 inhabitants in 2020 [22]. The areas of Ndorong and Parcelles Assainies were selected in Kaolack for the study (Fig 1).

## Larval habitat characterization

### Larval habitat selection

The study was carried out from August to December 2019 in the cities of Diourbel, Touba, and Kaolack to characterize malaria vector larval habitats, dynamics across rainy and dry seasons, and to identify the main larval ecology factors which may be contributing to urban malaria transmission. During the first survey carried out in August 2019, all encountered surface waters were visually inspected and confirmed as anopheline larval habitats when at least one immature stage (larvae or pupae) was found. During this first survey, a total of 145 larval habitats were identified, characterized and geo-localized for subsequent monthly monitoring. The prospected site included isolated pools, swamp margins, human-made ponds, natural ponds, drainage ditches, and human-made water basins. Once identified, larval habitats were geolocated, characterized, and larval indices were assessed. During the subsequent monthly surveys, all the recorded breeding sites were prospected to assess whether the presence or absence of anopheline immatures stages. Larval habitat GPS coordinates were collected using Open Data Kit (ODK) 24 resolution, accuracy less than 10m for mapping.

The habitat size was estimated in volume of water (cubic meters) as the product of the water surface (lengths by widths) and depth measured at the middle of the pool or drawn grid for the bigger water bodies.

### Larval survey data collection

Larval surveys were carried out monthly in each study city to monitor spatial and temporal variation. Each potential larval habitat was visually inspected to confirm the presence or absence of *Anopheles* larvae, and the habitat was considered as positive when at least at least one immature stage (larvae or pupae) was found, and negative when none was found. Both *Anopheles* and culicine larvae were identified to the genus level in each habitat, noting that *Anopheles* larvae are found parallel to the water surface while other genera have siphons and rest at an angle. Larvae were collected using a dipper of 350 ml, and when 10 or more larvae per dip were found, the larval density per liter of water was estimated by the dipping method using 10 dips for a total of 3.5 liters. For the smaller breeding sites, all larvae were collected, and the water collected was measured using a graduated container then the density was adjusted to 1 liter. All potential predators and non-anopheline organisms including other mosquito larvae were removed from the dipping collections and mosquito larvae were sorted as *Anopheles* or culicines based on the presence vs absence of the siphon (breathing tube) and their position to the water surface (anopheline larvae do not have a siphon and rest in parallel below the water surface, while culicine larvae have a siphon hang down and form an angle from the water surface). *Anopheles* larvae were then transferred to the insectary where they were reared to adulthood under controlled conditions (27˚C ± 2) for larvae and 25 ± 2°C and relative humidity of 70 ± 10% RH for adults that were morphologically identified using morphological identification keys [25,26] and further laboratory identification as needed using molecular techniques described by Scott et al. and Fanello et al. [27,28].

### Larval habitat classification

Larval habitats were classified using key parameters which included the type of the larval habitat (natural or anthropogenic), permanence (temporary or permanent), ecological and environmental profile, presence or absence of vegetation, and sunlight exposure (sunny or shaded). The larval habitats were considered permanent when they remained filled with water and contained *An*. *gambiae* s.l. larvae during all visits across the entire study period, while those that dried up before the end of the study period were considered as temporary/or non-permanent habitats.

### Larval habitat physico-chemical characteristics

The characterization of the physico-chemical parameters of larval habitats focused on the presence/absence of the vegetation, Ph, conductivity, salinity, and dissolved oxygen and were measured in a subset of surveyed larval habitats (n = 11) in Diourbel using a water analysis kit. This included ten permanent larval habitats and one productive temporary larval habitat at the Thierno Kandji station. However, the breakdown of the water analysis kit did not allow subsequent measures from the same habitats and from the others selected habitats in Diourbel as well as the two others study areas.

### Data analysis

The data were electronically captured in real time on a tablet using ODK collect and recorded in parallel in a field logbook then entered in an Excel database. Larval density was estimated from each positive habitat as the number of stage 3 and 4 *Anopheles* larvae per liter of water. When the volume of the collected water from a breeding site is less than or greater than one liter, the exact volume of the water collected from each site using the dipping method and count the total number of larvae divided by the exact volume then the density was adjusted to 1 liter of water. The source of the water, the size and depth of the larval habitat were recorded to estimate the ratio of larvae per estimated volume of the water in the larval habitat. Maps were drawn using the ArcMap (10.4) software, using the “Zone 28” on WGS84 UTM projection.

## Results

### Surveys of *An*. *gambiae* s.l. larval habitats

A total of 145 (32 in Diourbel, 83 in Touba and 30 in Kaolack) positive larval habitats were found and monitored monthly. Most of the *An*. *gambiae* s.l. larval habitats were found inside houses in artificial water storage basins in Touba, or in the immediate surroundings of human dwellings in Diourbel and Kaolack.

### City of Diourbel

**Fig 2:**
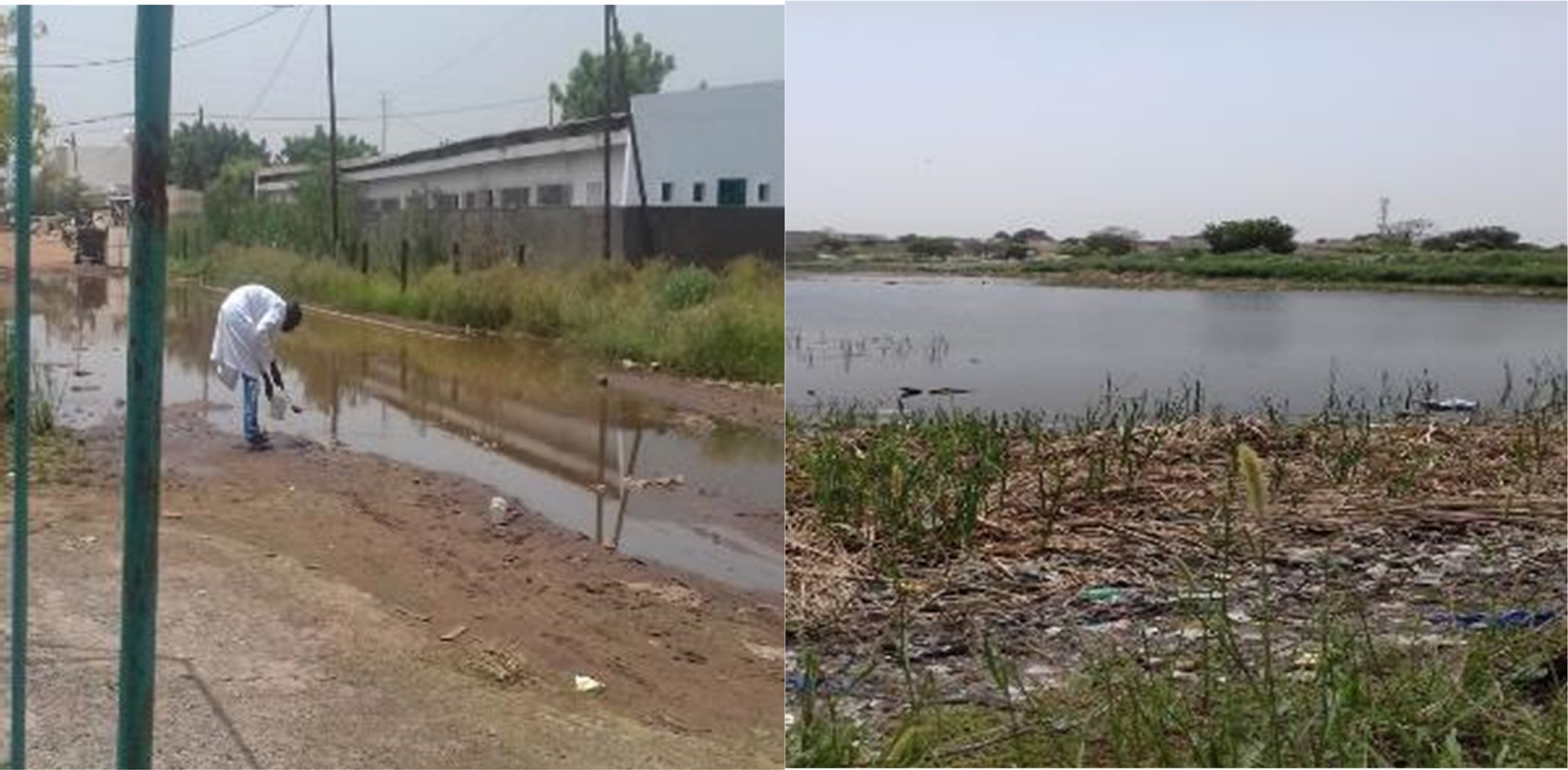
Flooding and larval habitats in Diourbel, Sénégal (Credit Fatou Ndiaye as Photographer).

In Diourbel, vector larval habitats consisted of natural water bodies (ponds and puddles) (37%) or anthropogenic habitats (puddles, borrow pits, depressions from road construction projects) (63%) (Fig 2). Additional larval habitats distanced from the compounds were also found in Diourbel, particularly at the periphery of the city.

**Fig 3:**
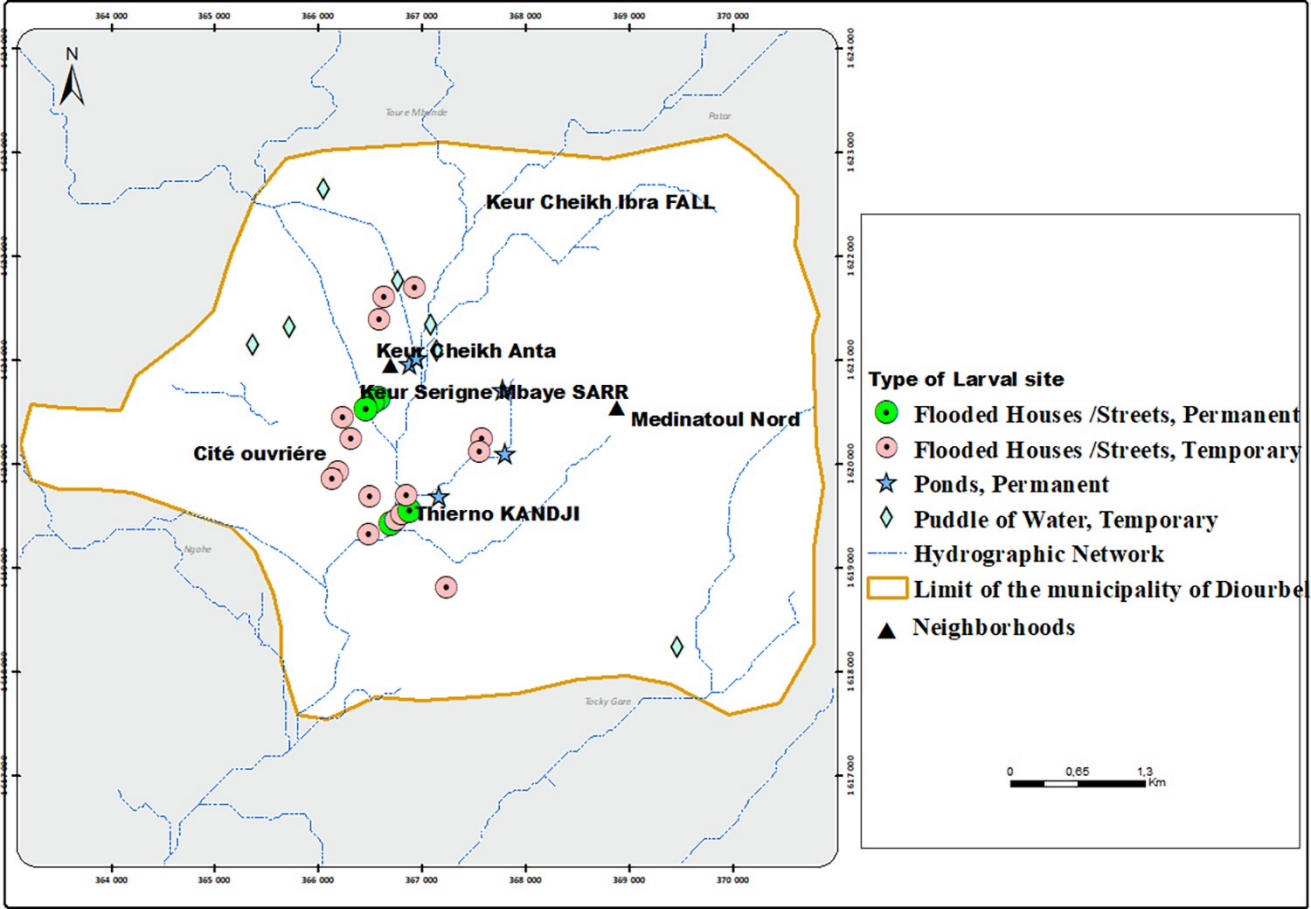
Spatial distribution of *An*. *gambiae* s.l. larval habitats in Diourbel, Sénégal (Copyright © 1995–2021 Esri. All rights reserved. Published in the United States of America.).

A total of 32 larval habitats including nine natural surface water bodies, 13 human-made, and 10 flooded areas and/or houses were monitored (Fig 3). The typology, the duration of water persistence and the spatial distribution of larval habitats revealed that they consisted mainly (68.75% (22/32)) of temporary habitats, lasting only during the rainy season. The temporary habitats all dried up by the end of the rainy season (November). However, about 31.25% (10/32) of habitats were found to be permanent larval habitats containing *An*. *gambiae* s.l. larvae throughout both the rainy and dry periods.

Most larval habitats in Diourbel were located in Keur Cheikh Anta (43.75%) and Thierno Kandji (34.37%), while few larval habitats (10%) were recorded in Keur Serigne Mbaye Sarr and its outskirts (Fig 2). Only one larval habitat consisting of a flooded, abandoned house was found and monitored in the Grand Diourbel station.

### City of Touba

**Fig 4:**
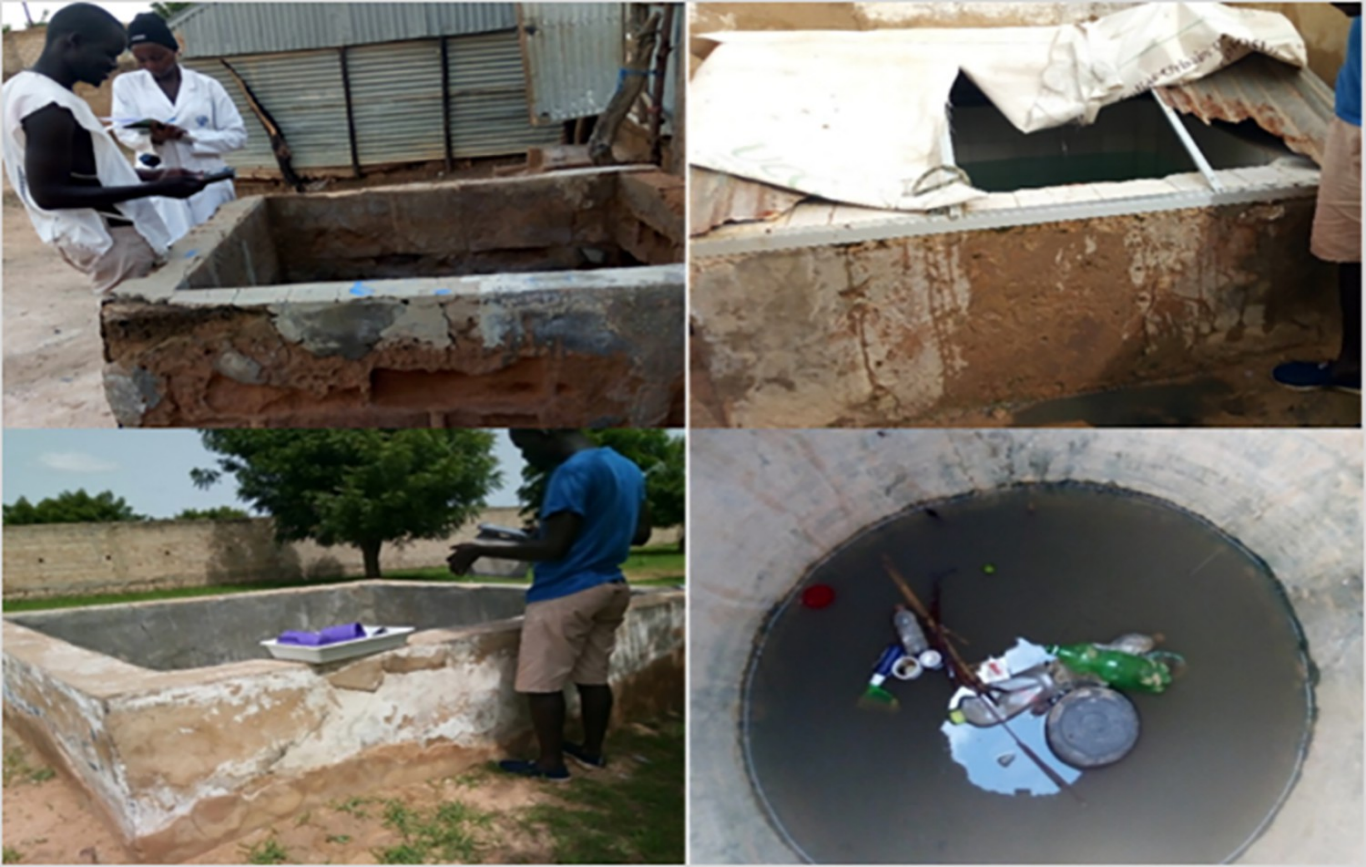
Example of water storage basins containing *An*. *gambiae* s.l. larvae in Touba (Credit Fatou Ndiaye as Photographer).

**Fig 5:**
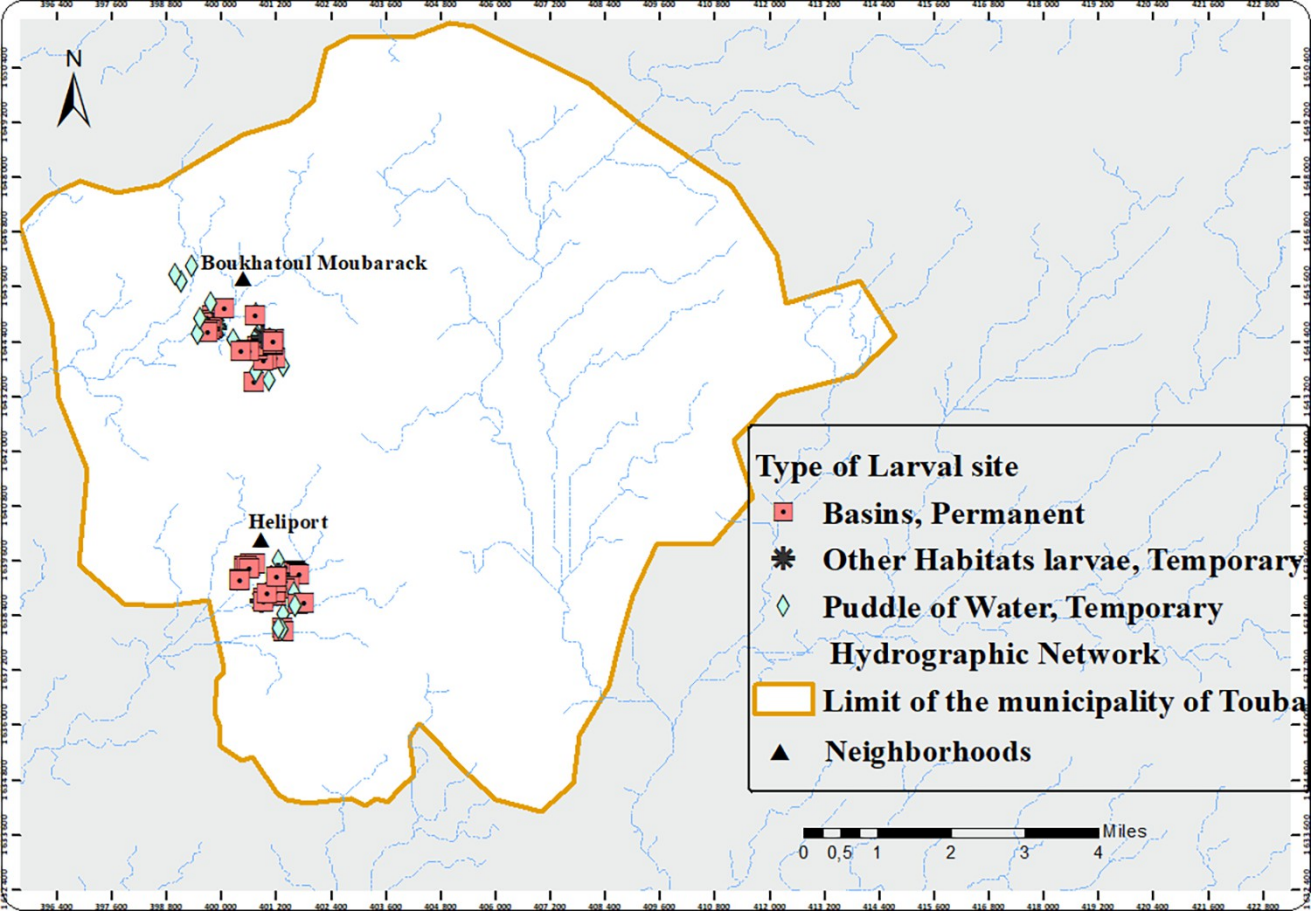
Spatial distribution of *An*. *gambiae* s.l. larval habitats in Touba, Sénégal (Copyright © 1995–2021 Esri. All rights reserved. Published in the United States of America.).

In Touba, artificial larval habitats were found and were mostly human-made water storage basins (66.3%) built inside housing compounds to store drinking water or water used for domestic activities such as laundry, bath, or for livestock, due to the scarcity of water in the city, especially during the dry season (Figs 4 & 5).

A total of 83 (55 basins and 28 temporary) habitats were monitored monthly throughout the study period.

The larval habitats surveyed were predominantly human-made water basins (66.3%, 55/83) followed by ponds (15.7%; 13/83) and puddles (9.6%; 8/83). The remaining 8.4% (7/83) were temporary larval habitats (flooded buildings under construction, human and animals’ footprints, and open septic tanks). The human-made water basin habitats in Boukhatoul Moubarak and Heliport were found exclusively inside and/or in the immediate surrounding areas of houses. By the end of December, only permanent human-made water basins (20.5%; 17/83) remained productive for *An*. *gambiae* s.l. in Touba. Of these, 10 were located in Heliport and seven in Boukhatoul Moubarack.

### City of Kaolack

**Fig 6:**
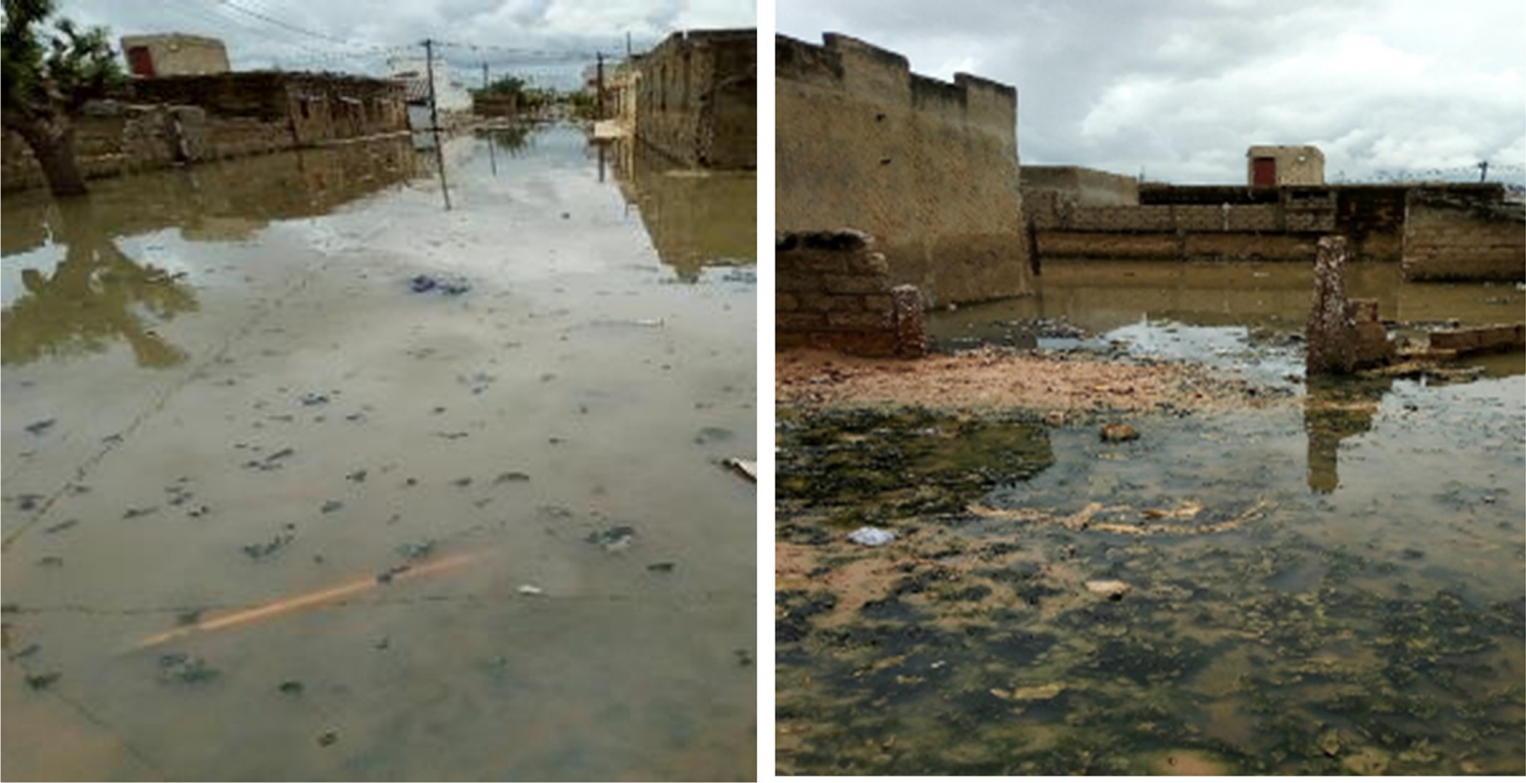
Flooding and larval habitats in Kaolack, Sénégal (Credit Fatou Ndiaye as Photographer).

**Fig 7:**
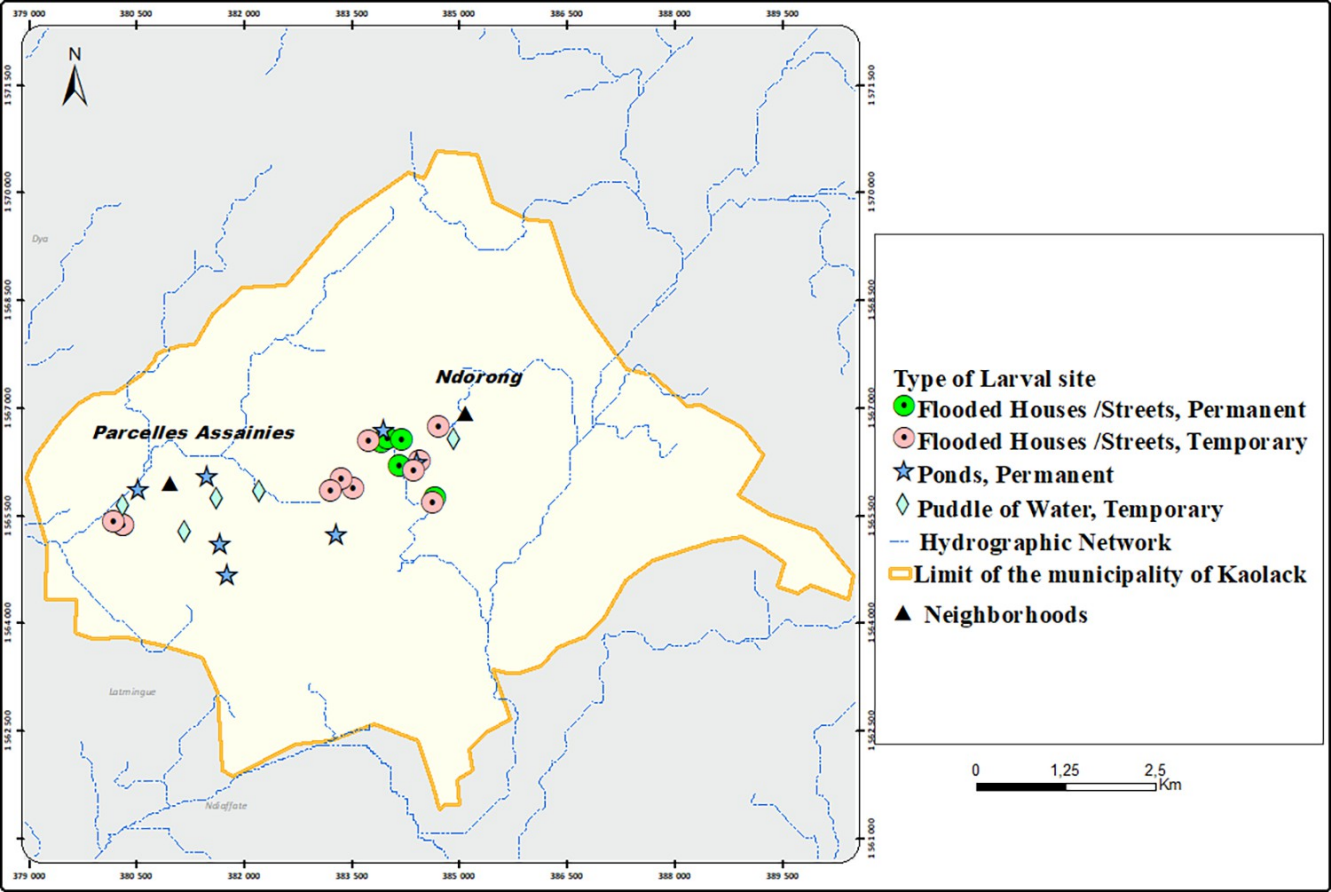
Spatial distribution of *An*. *gambiae* s.l. larval habitats in Kaolack, Sénégal (Copyright © 1995–2021 Esri. All rights reserved. Published in the United States of America.).

Kaolack was similar to Diourbel with larval habitats represented by natural water bodies or anthropogenic habitats. A total of 30 larval habitats, including 12 natural surface water bodies, 2 anthropogenic, and 16 flooded areas and / or houses (Figs 6 & 7) were found and monitored over the study period. The larval habitats were mainly anthropogenic and predominantly constituted by flooded areas and/or houses were more common in Ndorong, located in a lowland region. Natural breeding sites made of surface water bodies were more frequent in the Parcelles Assainies (66.7%, 8/12).

### Larval habitat characteristics

#### Type of vegetation

Where found, the larval habitats were mainly covered by submerged vegetation. Apart from bare wetlands which serve often as rainfed crop fields present in all cities, the presence of floating (algae) or standing vegetation (grass) was noted in most sites in Kaolack.

Vegetation was less frequently found in Touba given the nature and frequency of the usage of the water storage anthropogenic larval habitats. Only a few abandoned water storage basins contained floating algae.

#### Physico-chemical characteristic of larval habitat water in Diourbel

The mean pH of the larval habitat harboring *Anopheles* larvae was 7.9, with a minimum of 7.0 and maximum of 9.8. The mean conductivity and salinity were 9.3 ms and 0.5 g/l respectively while the mean oxygen found in the water bodies was 28.1 mg/l (Table 1).

**Table 1:** Chemical parameters of larval habitats found in the city of Diourbel.

Variable	pH	Conductivity (ms)	Salinity (g/l)	Dissolved oxygen (mg/l)
**Min**	6.95	1.654	0.08	19.6
**Max**	9.81	23.6	1.34	38.8
**Mean**	7.94	7.86	0.49	28.14
**Standard deviation**	± 0.7	± 4.17	± 0.23	± 6.14
**Confidence Interval**	[7.24–8.64]	[3.69–12.03]	[0.26–0.72]	[22–34.28]

#### Positivity and productivity rates of Anopheles larval habitats

**Fig 8:**
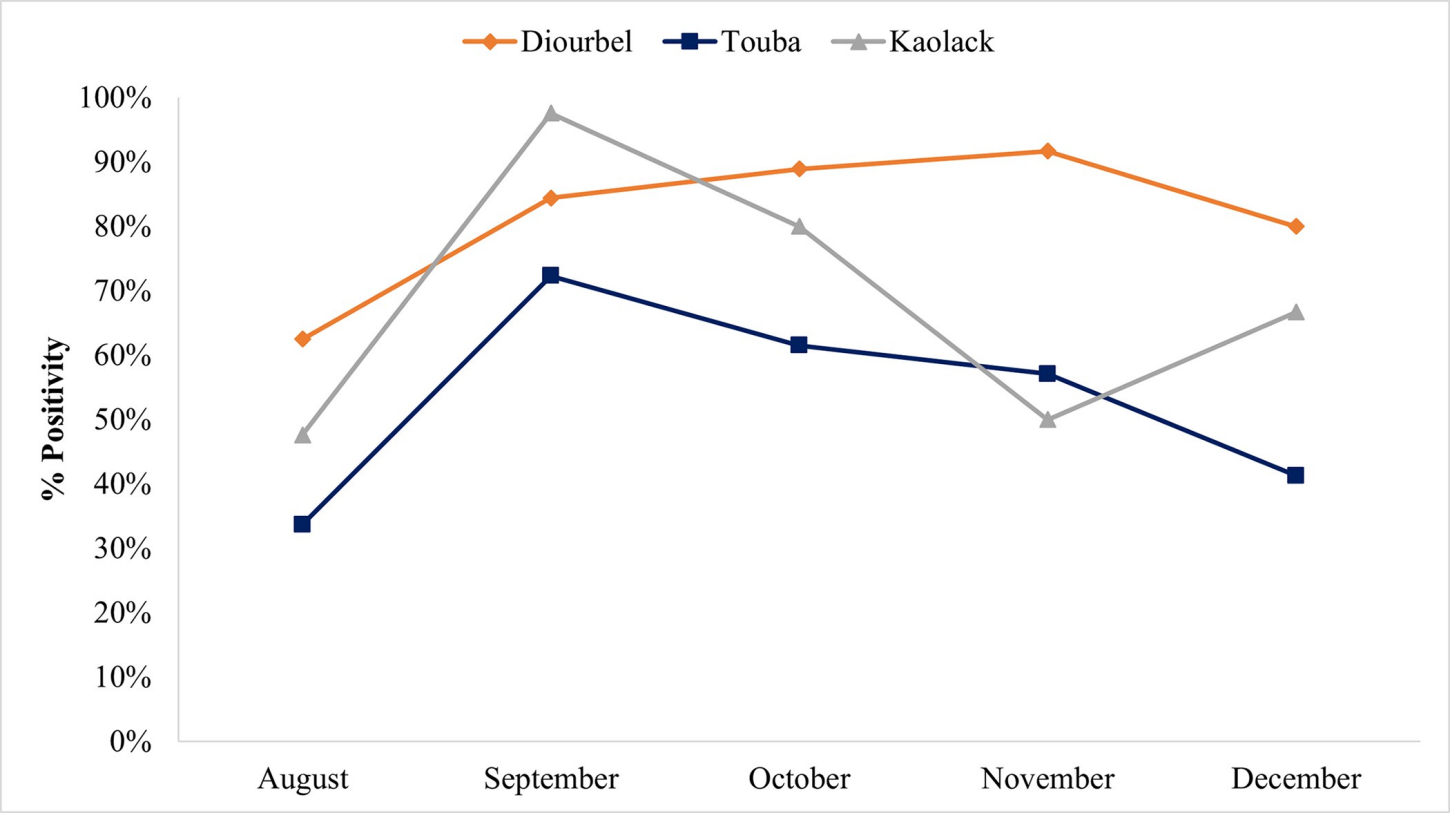
Monthly proportion of *An*. *gambiae* s.l. positive larval habitats in Diourbel, Touba and Kaolack out of the total surveyed monthly.

**Fig 9:**
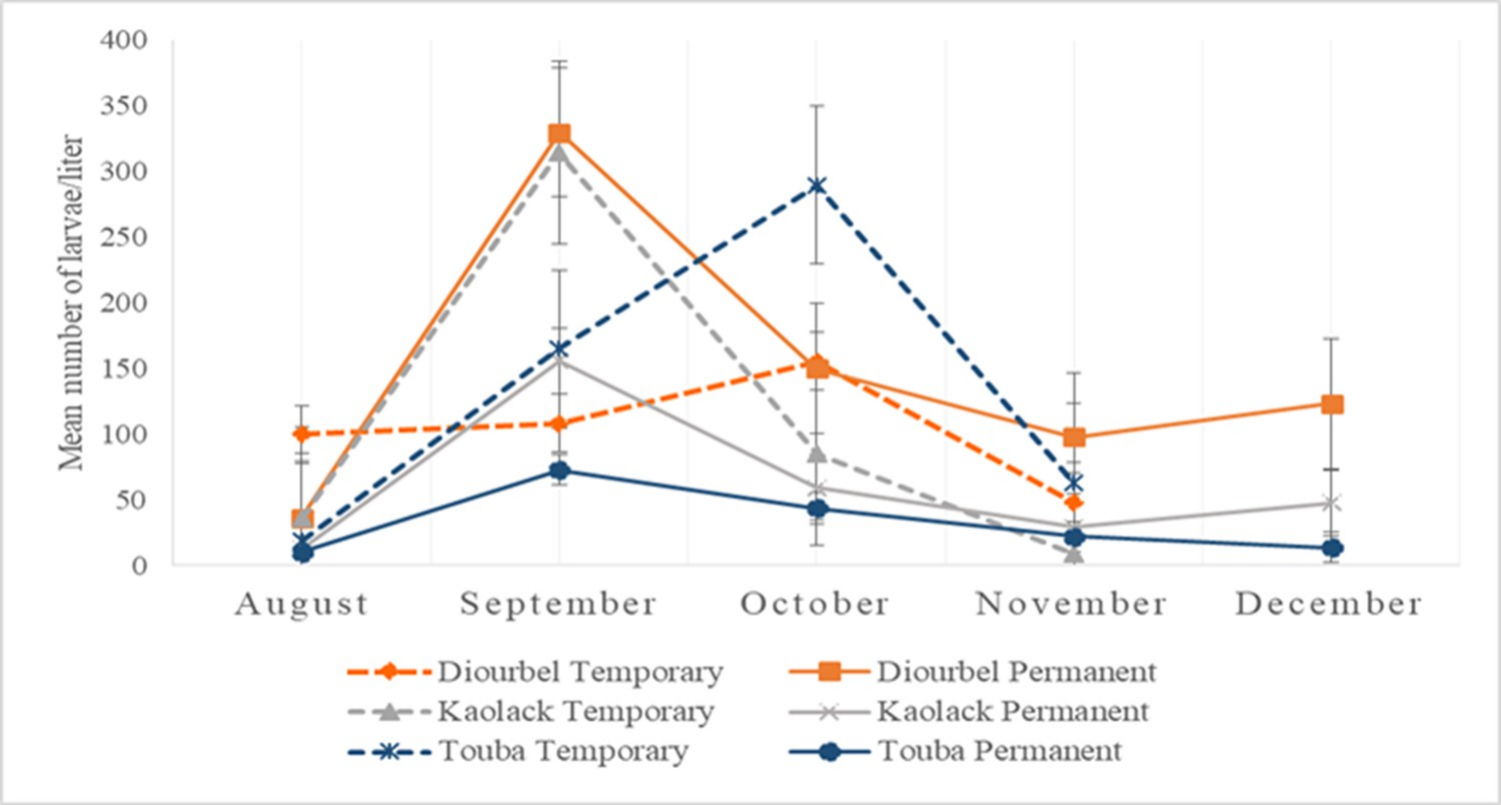
Trends in the mean number of *An*. *gambiae* s.l. larvae per larval habitat status in Diourbel, Touba and Kaolack.

The overall positivity rate of larval habitats was highest in Diourbel with the presence of at least one *Anopheles* larva in 27 out of the 32 sites monitored (84.4%) during the highest density in September 2019. The lowest rate was recorded in August 2019 (62.5%) (Fig 8). Furthermore, *An*. *gambiae* s.l. larvae were found in all the permanent sites through December (Fig 8). The peak larval density was recorded during the rainy season (August to October 2019). In Diourbel, permanent habitats were more productive in September 2019, with a peak of more than 300 larvae/liter compared to temporary habitats (mean of 155 larvae/liter) (Fig 9). By the end of the rainy season, mean larval densities dropped by 69% in the total remaining permanent habitats, while 68% (22/32) of the larval habitats dried out.

Conversely, the proportion of positive *An*. *gambiae* s.l. larval habitats was higher for temporary sites (290 larvae/liter) in Touba when compared to permanent sites during the highest larval density periods in September 2019, with positivity rates in temporary sites above 80% during these two months. However, as in Diourbel, the permanent larval habitats found in Touba persisted through December, and the temporary sites only persisted until November (Figs 8 & 9). Of all the temporary larval habitats found, 28 harboring *Anopheles* larvae were monitored. Water basins were the only type of permanent larval habitats detected in Touba throughout the study period as the natural larval habitats gradually dried up (57% in October, 71% in November) by December (100%). Noteworthy, basins were found dried up during previous surveys in a few areas but were refilled by tap water during subsequent visits. In Touba, at certain time points larval densities in the basins were similar to those found in the natural larval habitats. The average *An*. *gambiae* s.l. larval densities in the basins were in general less than 80 larvae / liter, except for a few basins constantly harboring higher larval densities during the whole study period (Table 2).

**Table 2:** Characteristics of the Larval habitats surveyed per district.

City	Types of larval habitats	Number	Type of habitat	Origin	Rainy Season		Dry Season		
					% Positivity	Mean Larval Density	% Positivity	Mean Larval Density	Remaining
**Diourbel**	Flooded Houses/Streets	**10** (31.3%)	Permanent	Natural	70–100%	330 larvae/liter	80%	123 larvae/liter	10
	Ponds								
	Flooded Houses/Streets	**22** (68.7%)	Temporary	Anthropophilic	59–75%	155 larvae/liter	Dried	Dried	Dried
	Puddle								
**Touba**	Basins	**55** (66.3%)	Permanent	Anthropophilic	40–56%	72 larvae/liter	43%	13 larvae/liter	17
	Ponds	**13** (15.7%)	Temporary	Natural	21–82%	290 larvae/liter	Dried	Dried	Dried
	Puddle	8 (9.6%)		Anthropophilic					
	Flooded buildings under construction	**3** (3.6%)							
	Open Septic Tanks	**1** (1.2%)							
	human and animal footprints	**3** (3.6%)							
**Kaolack**	Flooded Houses / Streets	**12** (40.0%)	Permanent	Natural	42–100%	156 larvae/liter	67%	48 larvae/liter	12
	Ponds								
	Flooded Houses / Streets	**18** (60.0%)	Temporary	Anthropophilic	50–70%	314 larvae/liter	Dried	Dried	Dried
	Puddle								

Of the 30 larval habitats selected and monitored in Kaolack, 12 (40%) were classified as permanent throughout the wet and dry season. The remaining 18 (60.0%) were classified as temporary habitats, all of which had dried up by November. The highest proportion of positive larval habitats was recorded during the rainy season when at least one *Anopheles* larva per liter was found in 97% and 80% of sites surveyed in September and October, respectively (Fig 8). The lowest proportion of positive larval habitats was noted in August for permanent (42%) and in November for the temporary larval habitats (25%). The lowest average larval densities were recorded in August (29 larvae / liter) and November (23 larvae / liter). The overall peak mean larval density was observed in September with 268 larvae / liter. During the rainy season, temporary sites were the most productive in larvae of *An*. *gambiae* s.l. (Fig 9), with more than 1,000 larvae / liter recorded in two of the 30 sites. However, permanent sites maintained larvae through December (Figs 8 & 9).

## Discussion

This is a preliminary study assessing the presence and type anopheline larval habitat in urban areas to inform additional studies and subsequent larval source management strategies. Also being limited to few selected neighborhoods, the study revealed widespread presence of permanent and productive malaria vector larval habitats across three urban areas in central Senegal. These results provide the first data on the characteristics, larval productivity, and seasonal permanence of anthropogenic *Anopheles* larval habitats in urban settings in the three selected cities. With increases in urban malaria presenting potential threats to malaria elimination in Senegal and the rising presence of urban malaria vector *An*. *stephensi* in Africa, this study highlights the heterogeneity in key productive larval habitats in different urban areas across rainy and dry seasons and similarities which could be used for decision making- all three urban areas have permanent *Anopheles* larval habitats which are productive across seasons and provide a reservoir for *Anopheles* larvae that is sustained even throughout dry periods. These findings provide entomological evidence of persistent malaria vector larval habitats in urban areas across rainy and dry periods which may be facilitating urban malaria transmission, and key larval habitat typologies which are the most productive. This information provides a greater understanding of potential drivers of urban malaria and entomological data which may support and guide the Senegal national malaria program to implement tailored supplementary interventions, such as larval source management (LSM), to accelerate national malaria elimination efforts, specifically in highly populated cities where urban malaria remains an important threat.

Larviciding and larval source management (LSM) has historically been an important strategy for malaria vector control. However, prior to its implementation, it is essential to identify, map, and characterize larval habitats where LSM may be feasible and most impactful and ensure that larval sites comply with WHO’s recommendation that treated sites are “Few, Fix and Findable” [29,30]. Urban areas are among the eligible settings where LSM is recommended to complement the core vector control interventions, such as ITNs, which may be a challenge to implement effectively in urban settings [31,32]. This study highlights dynamics, spatial and temporal distribution and persistence of larval habitats of the main malaria vector *An*. *gambiae* s.l. across three cities providing appropriate intervention timing indications.

Though the three selected cities have reported high urban malaria incidence (14.0 cases/1000 inhabitants in Touba, 9.2 cases per 1000 inhabitants in Diourbel and 8.7 cases/1000 inhabitants in Kaolack [22] in 2020), diverse larval habitats were observed, differentiating cities from one another suggesting the need for city specific control approaches. In Diourbel and Kaolack, the type and distribution of *An*. *gambiae* s.l. larval habitats were most similar and consisted mostly of flooded streets and houses in urban areas. This could be explained by ongoing urban development or inappropriate water drainage for rainwater evacuation, favoring water stagnation after rainfall suitable for *Anopheles* larvae [23]. These urban larval habitat features suggest the importance of coordinating control efforts across sectors, including those in urban development, to limit the establishment of mosquito larval habitats which may facilitate urban malaria transmission. Most human dwellings are built in fossil valleys that were originally wetlands in the peri-urban localities of Keur Serigne Mbaye Sarr in Diourbel and Parcelles Assainies in Kaolack, and in those sites most of the larval habitats consisted of rainfed surface water bodies, which correspond more to classical *An*. *gambiae* s.l. larval habitats [33]. In contrast to the other two cities, Touba most of the mosquito larval habitats were household human-made basins used for water storage. Furthermore, these larval habitats were found in more than 70% of human dwellings. Because these basins were unique to Touba, they limited the harmonization of larval habitat classification across sites; however, these habitats have the potential for LSM due to their permanence and it may be possible to sensitize the permanent and transient populations to manage water storage by removing or deconstructing unused basins and covering those that are still used. Furthermore, the persistence and use of the water basins was seasonally dependent particularly during the annual “Grand Magal” pilgrimage of Touba and other religious events where stored water was used and refilled almost daily. During other periods, water basins remain filled for several days or dried up without being refilled. Based on household needs, permanent larval habitats (basins) may require coverings or year-round management, while temporary larval habitats such as rain-filled ponds and flooded houses and streets may require more seasonal interventions, as they had largely dried up before the end of the study period. These findings differed slightly from Diedhiou *et al*.’s findings in Dakar suburbs which showed that flooded houses functioned as permanent productive larval habitats [23]; however, these differences could be attributed to differences in urban infrastructure in each city.

Diop *et al*. 2023, reported that most of the *An*. *gambiae* s.l. mosquitoes collected in the same three cities over the same period of time were *An*. *arabiensis*, and the species represented 100% of the mosquitoes collected in Touba [34]. The use of human-made water storage basins inside households and septic tanks under construction as larval habitats for *An*. *arabiensis* highlights the plasticity that this malaria vector has and its potential to drive transmission in urban areas where these habitats are abundant [35,36]. Though a considerable decrease in larval habitats and mean larval densities was observed during the months of November and December due to water shortages, the remaining basins containing water in Touba sustained and maintained *An*. *gambiae* s.l. populations, and therefore a certain level of malaria transmission risk throughout the dry season. This persistence of *Anopheles* larval habitats year-round in these urban areas is likely a contributing factor for malaria transmission stability and should call for integrated communication, monitoring and control activities. Furthermore, water storage basins which were shown here to be year-round productive *An*. *gambiae* s.l. habitats in Touba are also the types of habitats that have been found to be suitable for *An*. *stephensi*, an invasive urban malaria vector which has been shown to be associated with urban dry season malaria outbreaks in Africa [37–39]. Future larval surveys in urban Senegal similar should include molecular species identification of larvae to determine whether *An*. *stephensi* may have arrived in the country. Touba is a transportation and international trade hub which could facilitate the introduction of the invasive species to the country.

Larval habitats use, larval development, and survival often depend on the composition of species within the habitat and physicochemical properties of the water [38–41]. The physico-chemical characteristics of the larval habitats monitored in Diourbel fell within appropriate ranges to enable *Anopheles* vector larval development as described by different authors [42–44]. The results obtained showed that in Diourbel, alkaline larval habitats were the most common environments to find pre-imaginal stage *Anopheles* mosquitoes. It is known that pH and water oxygenation play an important role in mosquito development and may be related to the presence of vegetation [45–47]. In Diourbel, water oxygenation was strongly related to the presence of vegetation and the lowest oxygen levels were found in flooded houses of Keur Cheikh Anta, while the highest was recorded at Keur Serigne Mbaye Sarr where the presence of dense vegetation was observed. Similar to Keur Serigne Mbaye Sarr, high oxygen levels and low larval densities were recorded at Station 2 of Thierno Kandji surveyed larval habitats. The low pH and water oxygenation may have prevented high larval densities, since dissolved oxygen concentrations are often reported as negatively correlating with the abundance of *Anopheles* larvae [48,49]. These characteristics may be used to identify productive larval habitats in the future. One limitation of these findings is that the impact of the physico-chemical parameters of the water in the larval habitat was only carried out in the Diourbel, thus preventing comparison between the three surveyed cities, and representing a limitation of the study.

The study showed that across all three cities, the positivity and productivity of larval habitats showed heterogeneity and depended on the location, habitat type, and season. For example, in Diourbel and Kaolack, anthropogenic temporary larval habitats (puddles and flooded houses) were the predominant positive habitats during the rainy season (July, August and September) and yielded peaks of productivity during those months as previously reported in the country [8,49]. While permanent larval habitats in Diourbel and Kaolack consisted of natural water collections (pools and permanent surface water body in the fossil valley where larval habitats persist in riverbeds) producing *An*. *gambiae* s.l. larvae with high productivity, and maintaining vector populations, throughout the dry season. In contrast, in Touba, the permanent habitats were anthropogenic (human-made water basins), which allowed for *An*. *gambiae* s larval production to persist throughout the dry period. Despite heterogeneity, these findings suggest that in urban areas in Senegal, permanent malaria vector larval habitats exist, and these habitats should be identified and monitored to mitigate urban malaria transmission. These permanent habitats may also threaten the success of seasonal malaria control interventions in urban areas.

Another urban malaria study (Diop *et al*.) conducted in the same three cities in Senegal reported that the main population at risk of urban malaria is “Talibes”, or children attending traditional koranic schools known as “Daaras”, who are frequently exposed to vectors overnight during koranic learning hours and who sleep in limited spaces within schools or outdoors [34]. The larval habitat surveys described in the present study when combined with the Diop *et al*. vector biting and human exposure data may provide targeted solutions for urban malaria vector control, such as LSM, that would protect “Talibes" and the general population from urban malaria transmission.

## Conclusion

To tackle urban malaria in Senegal, this study provides a landscaping of malaria vector larval surveillance to better understand the key drivers of malaria transmission in urban settings. The results describe fixed, findable, geo-referenced *Anopheles* larval habitats along with the status of their permanence in three major cities, providing an opportunity to design and implement targeted LSM to supplement existing urban vector control interventions. Further, it highlights heterogeneity in key larval habitats across cities and the importance of considering urban infrastructure and water storage practices (particularly in Touba) to reduce *Anopheles* vector breeding sites and malaria transmission. Additionally, LSM activities could have the added benefit of limiting opportunities for other mosquito vectors to thrive, including *Aedes* spp. and invasive *An*. *stephensi*, in alignment with the WHO Global Vector Control Response strategy for integrated vector management. Continued and scaled up urban vector surveillance has the potential to enhance Senegal’s ability to use data driven decision making to respond to urban malaria, which threatens national malaria elimination efforts, and other mosquito borne diseases.

## References

[1] WHO: World malaria report. ISBN 978-92-4-004049-6 edition. pp. 322: World Health Organization; 2021:322.

[2] Bruce-Chwatt LJ: [Malaria and urbanization]. Bull Soc Pathol Exot Filiales 1983, 76:243–249. 6354493

[3] Donnelly MJ, McCall PJ, Lengeler C, Bates I, D’Alessandro U, Barnish G, Konradsen F, Klinkenberg E, Townson H, Trape JF, et al: Malaria and urbanization in sub-Saharan Africa. Malar J 2005, 4:12. doi: 10.1186/1475-2875-4-12 15720713 PMC552321

[4] Hay SI, Guerra CA, Tatem AJ, Atkinson PM, Snow RW: Urbanization, malaria transmission and disease burden in Africa. Nat Rev Microbiol 2005, 3:81–90. doi: 10.1038/nrmicro1069 15608702 PMC3130901

[5] Keiser J, Utzinger J, Caldas de Castro M, Smith TA, Tanner M, Singer BH: Urbanization in sub-saharan Africa and implication for malaria control. Am J Trop Med Hyg 2004, 71:118–127. 15331827

[6] Tatem AJ, Gething PW, Smith DL, Hay SI: Urbanization and the global malaria recession. Malar J 2013, 12:133. doi: 10.1186/1475-2875-12-133 23594701 PMC3639825

[7] Tatem AJ, Hay SI: Measuring urbanization pattern and extent for malaria research: a review of remote sensing approaches. J Urban Health 2004, 81:363–376. doi: 10.1093/jurban/jth124 15273262 PMC3173841

[8] Machault V, Gadiaga L, Vignolles C, Jarjaval F, Bouzid S, Sokhna C, Lacaux JP, Trape JF, Rogier C, Pagès F: Highly focused anopheline breeding sites and malaria transmission in Dakar. Malar J 2009, 8:138. doi: 10.1186/1475-2875-8-138 19552809 PMC2713260

[9] OECD, Sahel, Club WA: Africa&apos;s Urbanisation Dynamics 2020.2020.

[10] WHO: World malaria report 2022. Geneva, World Health Organization; 2022.

[11] Diallo A, Santos SD, Lalou R, Le Hesran JY: Perceived malaria in the population of an urban setting: a skipped reality in Dakar, Senegal. Malar J 2012, 11:340. 10.1186/1475-2875-11-340 PMC3507665 23043538

[12] Larson PS, Eisenberg JNS, Berrocal VJ, Mathanga DP, Wilson ML: An urban-to-rural continuum of malaria risk: new analytic approaches characterize patterns in Malawi. Malar J 2021, 20:418. doi: 10.1186/s12936-021-03950-5 34689786 PMC8543962

[13] Robert V, Macintyre K, Keating J, Trape JF, Duchemin JB, Warren M, Beier JC: Malaria transmission in urban sub-Saharan Africa. Am J Trop Med Hyg 2003, 68:169–176. 12641407

[14] Lines J, Harpham T, Leake C, Schofield C: Trends, priorities and policy directions in the control of vector-borne diseases in urban environments. Health Policy Plan 1994, 9:113–129. doi: 10.1093/heapol/9.2.113 15726774

[15] MSAS-DLM 2016. Plan Stratégique de lutte intégrée contre les maladies tropicales négligées 2016–2020. https://espen.afro.who.int/system/files/content/resources/SENEGAL_NTD_Master_Plan_2016_2020.pdf.

[16] Tangena JA, Hendriks CMJ, Devine M, Tammaro M, Trett AE, Williams I, DePina AJ, Sisay A, Herizo R, Kafy HT, et al: Indoor residual spraying for malaria control in sub-Saharan Africa 1997 to 2017: an adjusted retrospective analysis. Malar J 2020, 19:150. doi: 10.1186/s12936-020-03216-6 32276585 PMC7149868

[17] Thwing J, Eckert E, Dione DA, Tine R, Faye A, Yé Y, Ndiop M, Cisse M, Ndione JA, Diouf MB, Ba M: Declines in Malaria Burden and All-Cause Child Mortality following Increases in Control Interventions in Senegal, 2005–2010. Am J Trop Med Hyg 2017, 97:89–98. doi: 10.4269/ajtmh.16-0953 28990913 PMC5619933

[18] WHO: World malaria report. World Health Organization 2020, ISBN 978-92-4-001579-1:299.

[19] Garcia LS: Malaria. Clin Lab Med 2010, 30:93–129. 20513543 10.1016/j.cll.2009.10.001

[20] Lindsay SW, Birley MH: Climate change and malaria transmission. Ann Trop Med Parasitol 1996, 90:573–588. doi: 10.1080/00034983.1996.11813087 9039269

[21] Rossati A, Bargiacchi O, Kroumova V, Zaramella M, Caputo A, Garavelli PL: Climate, environment and transmission of malaria. Infez Med 2016, 24:93–104. 27367318

[22] NMCP: Bulletin epidemiologique annuel du paludisme au Senegal. pp. 612021:61.

[23] Diédhiou SM, Niang EA, Doucouré S, Samb B, Konaté A, Cissokho S, Ndiaye A, Wotodjo AN, Chauvancy G, Gadiaga L, et al: Distribution and characterization of anopheline larval habitats in flooded areas of the Dakar suburbs (Senegal). J Parasitol Vector Biol 2016, 8(7):61–73.

[24] Diédhiou SM, Konaté L, Doucouré S, Samb B, Niang EA, Sy O, Thiaw O, Konaté A, Wotodjo AN, Diallo M, et al: [Effectiveness of three biological larvicides and of an insect growth regulator against Anopheles arabiensis in Senegal]. Bull Soc Pathol Exot 2017, 110:102–115. 27942991 10.1007/s13149-016-0531-4

[25] Coetzee M: Key to the females of Afrotropical Anopheles mosquitoes (Diptera: Culicidae). Malar J 2020, 19:70. 10.1186/s12936-020-3144-9 PMC7020601 32054502

[26] Gillies MT, Coetzee M: A supplement to the Anophelinae of Africa south of the Sahara. Pub South Afr Inst for Med Res 1987, 55.

[27] Fanello C, Santolamazza F, and della Torre A: Simultaneous identification of species and molecular forms of the Anopheles gambiae complex by PCR-RFLP. Medical and Veterinary Entomology 2002. 16(4), 461–464. doi: 10.1046/j.1365-2915.2002.00393.x 12510902

[28] Scott JA, Brogdon WG, Collins FH, Scott JA, Brogdon WG, and Collins FH: Identification of single specimens of the Anopheles gambiae complex by the polymerase chain reaction 1993. 49(4). doi: 10.4269/ajtmh.1993.49.520 8214283

[29] Dambach P, Traoré I, Sawadogo H, Zabré P, Shukla S, Sauerborn R, Becker N, Phalkey R: Community acceptance of environmental larviciding against malaria with Bacillus thuringiensis israelensis in rural Burkina Faso—A knowledge, attitudes and practices study. Glob Health Action 2021, 14:1988279. doi: 10.1080/16549716.2021.1988279 34927578 PMC8725727

[30] Kamndaya M, Mfipa D, Lungu K: Household knowledge, perceptions and practices of mosquito larval source management for malaria prevention and control in Mwanza district, Malawi: a cross-sectional study. Malar J 2021, 20:150. doi: 10.1186/s12936-021-03683-5 33731146 PMC7967974

[31] Benelli G, Beier JC: Current vector control challenges in the fight against malaria. Acta Trop 2017, 174:91–96. doi: 10.1016/j.actatropica.2017.06.028 28684267

[32] WHO: Global framework for the response to malaria in urban areas. vol. ISBN 978-92-4-006178-1: World Health Organization, Geneva 2022.

[33] Minakawa N, Mutero CM, Githure JI, Beier JC, Yan G: Spatial distribution and habitat characterization of anopheline mosquito larvae in Western Kenya. Am J Trop Med Hyg 1999, 61:1010–1016. doi: 10.4269/ajtmh.1999.61.1010 10674687

[34] Diop A, Ndiaye F, Sturm-Ramirez K, Konate L, Senghor M, Diouf EH, Dia AK, Diedhiou S, Samb B, Sene D, Zohdy S, Dotson E, Diouf MB, Koscelnik V, Gerberg L, Bangoura A, Faye O, Clark T, Niang EHA, Chabi J. Urban malaria vector bionomics and human sleeping behavior in three cities in Senegal. Parasit Vectors. 2023 Sep 19;16(1):331. doi: 10.1186/s13071-023-05932-9 37726787 PMC10510207

[35] Hamza AM, El Rayah el A: A Qualitative Evidence of the Breeding Sites of Anopheles arabiensis Patton (Diptera: Culicidae) in and Around Kassala Town, Eastern Sudan. Int J Insect Sci 2016, 8:65–70. doi: 10.4137/IJIS.S40071 27547039 PMC4982522

[36] Azrag RS, Mohammed BH: Anopheles arabiensis in Sudan: a noticeable tolerance to urban polluted larval habitats associated with resistance to Temephos. Malar J 2018, 17:204. doi: 10.1186/s12936-018-2350-1 29776357 PMC5960190

[37] Balkew M, Mumba P, Dengela D, Yohannes G, Getachew D, Yared S, Chibsa S, Murphy M, George K, Lopez K, et al: Geographical distribution of Anopheles stephensi in eastern Ethiopia. Parasit Vectors 2020, 13:35. doi: 10.1186/s13071-020-3904-y 31959237 PMC6971998

[38] Balkew M, Mumba P, Yohannes G, Abiy E, Getachew D, Yared S, Worku A, Gebresilassie A, Tadesse FG, Gadisa E, et al: An update on the distribution, bionomics, and insecticide susceptibility of Anopheles stephensi in Ethiopia, 2018–2020. Malar J 2021, 20:263. doi: 10.1186/s12936-021-03801-3 34107943 PMC8189708

[39] Sinka ME, Pironon S, Massey NC, Longbottom J, Hemingway J, Moyes CL, Willis KJ: A new malaria vector in Africa: Predicting the expansion range of Anopheles stephensi and identifying the urban populations at risk. Proc Natl Acad Sci U S A 2020, 117:24900–24908. doi: 10.1073/pnas.2003976117 32929020 PMC7547157

[40] Getachew D, Balkew M, Tekie H: Anopheles larval species composition and characterization of breeding habitats in two localities in the Ghibe River Basin, southwestern Ethiopia. Malar J 2020, 19:65. doi: 10.1186/s12936-020-3145-8 32046734 PMC7014609

[41] Low M, Tsegaye AT, Ignell R, Hill S, Elleby R, Feltelius V, Hopkins R: The importance of accounting for larval detectability in mosquito habitat-association studies. Malar J 2016, 15:253. doi: 10.1186/s12936-016-1308-4 27142303 PMC4855760

[42] Gimnig JE, Ombok M, Kamau L, Hawley WA: Characteristics of larval anopheline (Diptera: Culicidae) habitats in Western Kenya. J Med Entomol 2001, 38:282–288. doi: 10.1603/0022-2585-38.2.282 11296836

[43] Kweka EJ, Zhou G, Lee MC, Gilbreath TM 3rd, Mosha F, Munga S, Githeko AK, Yan G: Evaluation of two methods of estimating larval habitat productivity in western Kenya highlands. Parasit Vectors 2011, 4:110. 21682875 10.1186/1756-3305-4-110 PMC3138440

[44] Ndenga BA, Simbauni JA, Mbugi JP, Githeko AK, Fillinger U: Productivity of malaria vectors from different habitat types in the western Kenya highlands. PLoS One 2011, 6:e19473. doi: 10.1371/journal.pone.0019473 21559301 PMC3085476

[45] Mogi M, Okazawa T, Miyagi I, Sucharit S, Tumrasvin W, Deesin T, Khamboonruang C: Development and survival of anopheline immatures (Diptera: Culicidae) in rice fields in northern Thailand. J Med Entomol 1986, 23:244–250. doi: 10.1093/jmedent/23.3.244 3735328

[46] Parham PE, Pople D, Christiansen-Jucht C, Lindsay S, Hinsley W, Michael E: Modeling the role of environmental variables on the population dynamics of the malaria vector Anopheles gambiae sensu stricto. Malar J 2012, 11:271. doi: 10.1186/1475-2875-11-271 22877154 PMC3496602

[47] Dida GO, Anyona DN, Abuom PO, Akoko D, Adoka SO, Matano AS, Owuor PO, Ouma C: Spatial distribution and habitat characterization of mosquito species during the dry season along the Mara River and its tributaries, in Kenya and Tanzania. Infect Dis Poverty 2018, 7:2. doi: 10.1186/s40249-017-0385-0 29343279 PMC5772712

[48] Mereta ST, Yewhalaw D, Boets P, Ahmed A, Duchateau L, Speybroeck N, Vanwambeke SO, Legesse W, De Meester L, Goethals PL: Physico-chemical and biological characterization of anopheline mosquito larval habitats (Diptera: Culicidae): implications for malaria control. Parasit Vectors 2013, 6:320. doi: 10.1186/1756-3305-6-320 24499518 PMC4029358

[49] Hawaria D, Demissew A, Kibret S, Lee MC, Yewhalaw D, Yan G: Effects of environmental modification on the diversity and positivity of anopheline mosquito aquatic habitats at Arjo-Dedessa irrigation development site, Southwest Ethiopia. Infect Dis Poverty 2020, 9:9. doi: 10.1186/s40249-019-0620-y 31987056 PMC6986026

